# Unveiling the Larvicidal Potential of Golpar (*Heracleum persicum* Desf. ex Fisch.) Essential Oil and Its Main Constituents on *Aedes* and *Anopheles* Mosquito Vectors

**DOI:** 10.3390/plants13212974

**Published:** 2024-10-24

**Authors:** Marta Ferrati, Cecilia Baldassarri, Paolo Rossi, Guido Favia, Giovanni Benelli, Livia De Fazi, Mohammad Reza Morshedloo, Luana Quassinti, Riccardo Petrelli, Eleonora Spinozzi, Filippo Maggi

**Affiliations:** 1 Chemistry Interdisciplinary Project (ChIP), School of Pharmacy, University of Camerino, Via Madonna delle Carceri, 62032 Camerino, Italy; marta.ferrati@unicam.it (M.F.); riccardo.petrelli@unicam.it (R.P.); filippo.maggi@unicam.it (F.M.); 2 School of Biosciences and Veterinary Medicine, University of Camerino, Via Gentile III Da Varano, 62032 Camerino, Italy; cecilia.baldassarri@unicam.it (C.B.); paolo.rossi@unicam.it (P.R.); guido.favia@unicam.it (G.F.); 3 Department of Agriculture, Food and Environment, University of Pisa, Via del Borghetto 80, 56124 Pisa, Italy; giovanni.benelli@unipi.it (G.B.); livia.defazi@phd.unipi.it (L.D.F.); 4 Department of Horticultural Science, Faculty of Agriculture, University of Maragheh, Maragheh 551877684, Iran; morshedloo110@gmail.com; 5 School of Pharmacy, University of Camerino, Via Gentile III Da Varano, 62032 Camerino, Italy; luana.quassinti@unicam.it

**Keywords:** Apiaceae, *Aedes aegypti*, *Aedes albopictus*, *Anopheles gambiae*, octyl acetate, hexyl butyrate, insecticide

## Abstract

Natural products are thoroughly studied as valuable alternatives to synthetic insecticides. *Heracleum persicum* Desf. ex Fisch. (Apiaceae), commonly known as Golpar, is an Iranian medicinal plant largely employed as a spice, which has previously revealed insecticidal potential. The chemical composition of *H. persicum* essential oil (EO) was investigated by GC-MS and was mainly dominated by hexyl butyrate (36.1%) and octyl acetate (23.7%). The EO and its main esters were tested on three mosquito species. *Aedes aegypti* (L.) larvae were the most sensitive to all tested products. Lethal concentrations (LC_50_) of 59.09, 53.59, and 47.05 ppm were recorded for the EO, hexyl butyrate, and octyl acetate, respectively. *Aedes albopictus* (Skuse) and *Anopheles gambiae* Giles demonstrated comparable sensitivity to the EO, with LC_50_ values of 102.97 and 97.91 ppm, respectively, whereas the isolated constituents appeared more active on *An. gambiae* (LC_50_ of hexyl butyrate and octyl acetate of 70.97 and 60.71 ppm, respectively) with respect to *Ae. albopictus* (LC_50_ of hexyl butyrate and octyl acetate of 85.40 and 91.38 ppm, respectively). Low toxicity was registered for both EO and single components against human embryonic kidney (HEK293) cells. Overall, the *H. persicum* EO, hexyl butyrate, and octyl acetate could be further considered for larvicide development.

## 1. Introduction

The rapid and continuous emergence of vector-borne diseases (VBDs) represents one of the major causes of public health concern worldwide [1,2]. VBDs are linked to 17% of the estimated burden of infectious diseases and are caused by pathogens spread by arthropods, such as sand flies, tsetse flies, lice, triatomine bugs, ticks, and mosquitoes [3,4]. The latter transmit dangerous pathogens that cause dengue, malaria, chikungunya, yellow fever, West Nile virus, Zika virus, and filariasis, among others [5,6]. A key tool for managing mosquito-borne diseases is vector control, consisting in the reduction or elimination of vector–host interactions and consequently limiting pathogen transmission [7,8]. Among mosquito management programs, chemical control tools are quite common [8], but their uncontrolled employment often leads to negative effects on human health and the environment [9,10,11], as well as the continuous onset of insecticide resistance [9,12,13].

For these reasons, regulatory authorities are searching for innovative, harmless, and eco-friendly alternatives, and botanicals are increasingly gaining more attention [14,15,16]. Among them, essential oils (EOs), complex mixtures of volatile compounds, have demonstrated promising potential against several insect vectors and pests [16,17]. Besides their proven bioactivity, their multiple modes of action reduce the likelihood of resistance phenomena [18]. Furthermore, EOs are characterized by low toxicity to non-targets and limited persistence in the environment. Lastly, these products are usually industrially employable due to the large supply of raw materials for food, flavorings and fragrances, and cosmetics [19]. This observation drives the motivation to explore botanical sources for their insecticidal potential. In this context, *Heracleum persicum* Desf. ex Fisch. (Apiaceae) is a perennial flowering plant native to Iran, Turkey, and Iraq, where it is also known as Golpar or Persian hogweed [20]. This plant grows up to 50–120 cm and has red-brown stems with alternate leaves, while the blades are long, pinnate, and glabrous on the top. Golpar produces small white/green flowers and obovate fruits [21]; it is widely used as a spice in Persian cuisine and perfumery, also being extensively used for the treatment of respiratory, neurological, urinary, gastrointestinal, and rheumatological disorders [22]. The plant is distributed in Iran and, due to the high demand, also largely cultivated in the north of the country. Indeed, it requires well-drained soils with full exposure to sunlight and regular watering and fertilization. Golpar can be easily propagated through seeds or by dividing existing plants. The price of schizocarps in Iran is 2–3 USD/kg.

The insecticidal potential of *H. persicum* has also been reported, especially that of its EO, which is composed of aliphatic esters, mainly hexyl butyrate and octyl acetate. The EO showed toxic effects on the mosquito *Anopheles stephensi* Liston [23] and on stored product pests, such as *Callosobruchus maculatus* (F.) [24] and *Tribolium castaneum* (Herbst) [25]. Regarding the main constituents, Baranová et al. [26] recently demonstrated that octyl acetate was even more effective than *Heracleum mantegazzianum* Sommier & Levier EO against *Aedes japonicus* Theobald (LC_50_ values of 67 mg/L and 52 mg/L for the EO and octyl acetate, respectively).

This study focused on EO obtained from *H. persicum* schizocarps, hexyl butyrate and octyl acetate, evaluating them as insecticides against larvae of *Aedes aegypti* (L.), the yellow fever mosquito, *Aedes albopictus* (Skuse), vector of several arboviruses and lymphatic filariasis, and *Anopheles gambiae* Giles, the major vector of malaria in sub-Saharan Africa [5,27]. Furthermore, the products were tested on human non-tumoral embryonic kidney 293 cells (HEK293) to evaluate their toxicity.

## 2. Results and Discussion

### 2.1. Essential Oil (EO) Chemical Analysis

*H. persicum* schizocarps EO was characterized mainly by aliphatic compounds, with esters being the most abundant class (87.9%). Alcohols were present in minor amounts (5.9%), followed by aldehydes (0.6%) (Table 1).

Among esters, hexyl butyrate and octyl acetate were the predominant ones (36.1 and 23.7% of the total composition, respectively).

*H. persicum* EO’s composition mostly depends on the organs from which the EO is extracted and on the different collection stages of the plant [31]. Usually, EOs from leaves, stems, and fruits are mainly constituted by terpenoids such as (*E*)-anethole, *β*-ocimene, and *β*-pinene and only to a small extent by aliphatic compounds [32,33]. On the other hand, EOs obtained through the distillation of ripe or unripe seeds are mainly characterized by the presence of aliphatic esters and aldehydes [25,34,35,36].

These observations are consistent with the results presented here. The schizocarps from which the EO was obtained were collected at the full ripening stage in July and yielded a product rich in aliphatic esters, particularly hexyl butyrate (36.1%) and octyl acetate (23.7%). Generally, studies conducted on different varieties and ripening stages of *H. persicum* showed that the production of these aliphatic compounds occurs in almost all the varieties in the post-flowering stages of the plant [31]. Moreover, different amounts of esters and aldehydes seem to depend on the soil, environment, and geographic area of cultivation. Radjabian et al. [37] highlighted the existence of three main chemotypes of *H. persicum* distributed across 17 different Iranian geographic areas, categorized based on the concentrations of the two most abundant esters. The first group is characterized by equal amounts of hexyl butyrate and octyl acetate, while the second contains more octyl acetate than hexyl butyrate. The third group, to which the plant material analyzed in this study belongs to, has a higher amount of hexyl butyrate compared with octyl acetate [37]. Hasani et al. [38] also demonstrated that the difference in concentration of hexyl butyrate, octyl acetate, and other aliphatic compounds (hexyl isobutyrate, octenol acetate, hexyl-2-methyl butyrate, octyl isobutyrate, hexyl hexanoate, *n*-octyl butyrate, and *n*-octyl-2-methyl butyrate) can be influenced by the concentration of salts and nitrogen in the soil on which the plant grows [38]. The presence of aliphatic esters is limited in taxonomic distribution among plant species and has been mostly reported for EOs from plant species belonging to the genus *Heracleum* L., such as *H. sphondylium* L., *H. gorganicum* Rech.f., *H. rechingeri* Manden, *H. anisactis* Boiss. & Hohen, *H. pastinacifolium* K.Koch, and *H. rawianum* C.C.Towns [39,40,41,42]. The above-mentioned compounds are correlated with each other from a phenotypical base but also with bergapten and xanthotoxin, which are phototoxic furanocoumarins co-occurring in the vittae of the plants where the esters are produced [43]. The latter probably act as carrier solvents enhancing the diffusion of the furanocoumarins into integuments and gut walls of herbivores [44].

### 2.2. Mosquito Larvicidal Assays

Median lethal concentrations (LC_50_) on *Ae. albopictus* were 102.97, 85.40, and 91.38 ppm, while concentrations able to kill 90% of the exposed larvae (LC_90_) were 122.4, 113.65, and 122.22 ppm for the EO, hexyl butyrate, and octyl acetate, respectively (Table 2).

**Figure 1:**
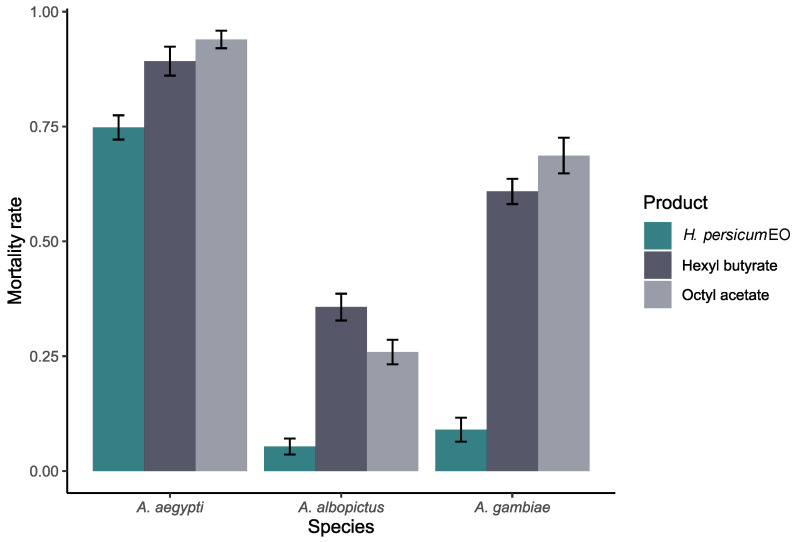
Mortality rate of *Aedes aegypti*, *Aedes albopictus*, and *Anopheles gambiae* larvae when exposed to *Heracleum persicum* essential oil (EO), hexyl butyrate, and octyl acetate.

**Figure 2:**
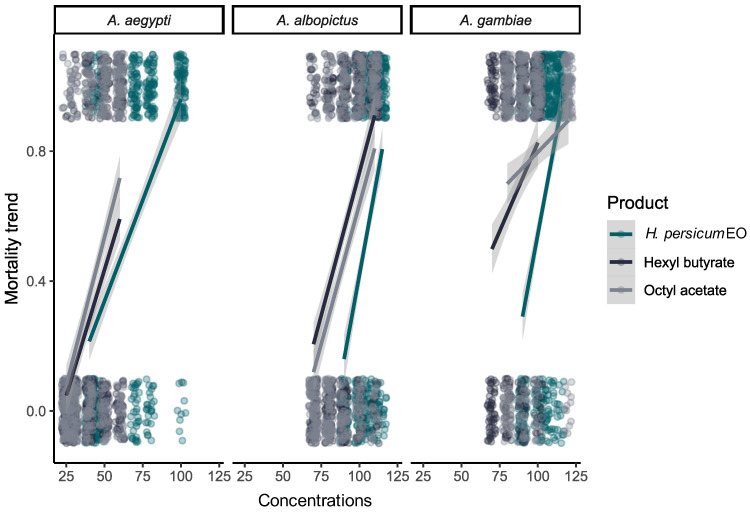
Mortality trend of *Aedes aegypti*, *Aedes albopictus*, and *Anopheles gambiae* by increasing the dose of *Heracleum persicum* essential oil (EO), hexyl butyrate, and octyl acetate.

Both hexyl butyrate and octyl acetate were more effective against this species than the EO (GLMM post hoc Bonferroni corrected—hexyl butyrate: OR = 0.101, SE = 0.037, z = −6.265, *p* < 0.0001; octyl acetate: OR = 0.161, SE = 0.059, z = −4.935, *p* < 0.0001) (Figure 1). Even if no significant difference between the two compounds was detected (OR = 1.588, SE = 0.300, z = 2.448, *p* = 0.129), the LC_50_ and LC_90_ were lower for hexyl butyrate if compared with octyl acetate (Figure 2).

LC_50_ on *Ae. aegypti* was 59.09, 53.59, and 47.05 ppm, while the LC_90_ was 101.62, 99.49, and 84.30 ppm for the EO, hexyl butyrate and octyl acetate, respectively (Table 2). Also in this case, hexyl butyrate and octyl acetate were more toxic than the EO (hexyl butyrate: OR = 0.357, SE = 0.128, z = −2.874, *p* = 0.036; octyl acetate: OR = 0.191, SE = 0.191, z = −4.555, *p* < 0.0001) (Figure 1). As above, no difference was detected between the two compounds for the mortality (OR = 0.534, SE = 0.251, z = −1.333, *p* = 1.000), but octyl acetate had lower LC_50_ and LC_90_ values with respect to hexyl butyrate (Figure 2).

LC_50_ on *An. gambiae* was 97.91, 70.97, and 60.71 ppm, while the LC_90_ was 116.02, 116.48, and 125.45 ppm for the EO, hexyl butyrate, and octyl acetate, respectively (Table 2). Again, both hexyl butyrate and octyl acetate were more effective against this species than the EO (hexyl butyrate: OR = 0.063, SE = 0.021, z = −8.116, *p* < 0.0001; octyl acetate: OR = 0.045, SE = 0.016, z = −8.449, *p* < 0.0001) (Figure 1), even if no significant difference was detected between these two compounds (OR = 0.709, SE = 0.151, z = −1.604, *p* = 0.978). Nevertheless, although the two compounds were found to be more effective than the EO, their efficacy is slower; even a slight increase in EO concentration leads to a higher mortality rate, whereas this is not as true for hexyl butyrate and octyl acetate. Thus, their LC_50_ and LC_90_ trends are significantly different from the EO ones (EO vs. hexyl butyrate: OR = 0.072, SE = 0.016, z = 4.372, *p* = 0.0001; EO vs. octyl acetate: OR = 0.092, SE = 0.016, z = 5.731, *p* < 0.0001) (Figure 2).

All the assays with *H. persicum* EO and its two major constituents showed the larvicidal potential of the tested products. A comparison of LC_50_ and LC_90_ values reveals that the concentrations required to affect *Ae. albopictus* and *An. gambiae* were significantly higher than those needed for *Ae. aegypti*, indicating the heightened susceptibility of *Ae. aegypti* to *H. persicum* EO. This increased sensitivity may be due to the different anatomical and physiological characteristics of this species. This finding is particularly striking given the close phylogenetic relationship between *Ae. albopictus* and *Ae. aegypti*. However, a comprehensive understanding of the mechanisms underlying the different sensitivity remains challenging.

Usually, botanical products with a LC_50_ lower than 100 ppm can be considered interesting for their application as insecticidal agents [45,46]. The EO herein investigated achieved LC_50_ values lower than 100 ppm against *An. gambiae* and *Ae. aegypti* and slightly higher against *Ae. albopictus*. Interestingly, hexyl butyrate and octyl acetate showed significantly higher efficacy towards larvae of all mosquito species. Indeed, they could be mainly responsible for the effect of the EO. Considering the few reports regarding the mosquitocidal activity of EOs of the *Heracleum* genus, the presented results are of interest. Indeed, they could be useful to better assess the insecticidal potential of this Iranian plant. This study reports for the first time the larvicidal effects of *H. persicum* EO on *Ae. aegypti*, *An. gambiae* and *Ae. albopictus*. Previously, only Sedaghat et al. [23] assayed the EO from seeds of the same species on fourth instar larvae of *An. stephensi*, yielding comparable results to those reported in this study for mosquitoes of the *Anopheles* genus. The product showed moderate toxicity, with LC_50_ and LC_90_ values of 104.80 and 174.22 ppm, respectively, after 24 h of exposure. Unfortunately, the chemical composition of the EO has not been reported in that study. The insecticidal activity of the EO against agricultural pests has also been shown. Specifically, it showed toxicity against adults of *C. maculatus* and the sub-lethal doses of this EO reduced the longevity and fecundity of the beetle, also reducing female fertility by 21.2%, with a significant effect on the oviposition behavior [47]. Moreover, Manzoomi et al. [24] reported that the fumigant toxicity of this EO increased at increasing concentrations and exposure time (LC_50_ of 337.58 μL/L) against the same species. Furthermore, *H. persicum* EO exhibited strong repellent effects also against *T. castaneum*. Other species of the genus *Heracleum* showed toxicity against mosquitoes. For instance, Baranová et al. [26] recently tested *H. mantegazzianum* EO against *Ae. japonicus* Theobald larvae. Its chemical composition was dominated by octyl acetate (58.65% of the total identified), which was also more efficient than the EO (LC_50_ values of 67 mg/L and 52 mg/L for the EO and octyl acetate, respectively). Moreover, Govindarajan et al. [48] showed high toxicity of the *Heracleum sprengelianum* Wight & Arn. EO against larvae of *Anopheles subpictus* Grassi, a malaria vector, *Ae. albopictus*, and the Japanese encephalitis vector *Culex tritaeniorhynchus* Giles, obtaining LC_50_ of 33.4, 37.5, and 40.9 mg/mL, respectively. The main EO components were lavandulyl acetate and bicyclogermacrene, which exhibited even lower larval toxicity. The LC_50_ values ranged from 4.17 to 10.3 mg/mL against *An. subpictus*, 4.60 to 11.1 mg/mL against *Ae. albopictus*, and 5.11 to 12.5 mg/mL against *C. tritaeniorhynchus*. Finally, the acute toxicity of the EO obtained from *H. sphondylium* subsp. *sphondylium* and *H. sphondylium* subsp. *ternatum* also showed efficacy on third instar larvae of the filariasis vector *Culex quinquefasciatus* Say, reaching LC_50_ values of 73.8 and 64.98 μL/L, respectively [44]. Based on the results herein obtained and those reported in the literature, it is worth continuing to investigate the EOs of the *Heracleum* genus for their exploitation as eco-friendly insecticides. Particularly, the mechanism(s) of action [18] and the non-target toxicity [6] of these botanical products should be assessed. Aliphatic esters already demonstrated to have a toxic behavior on the moth *Depressaria radiella* (Duponchel, 1838) which produces specific esterases involved in the detoxification system [49]. However, no data are available on the larvicidal mode of action of hexyl butyrate and octyl acetate. Nonetheless, the low LC_50_ values obtained, especially on *Ae. aegypti*, highlight the possible role of these products in mosquito management. Additionally, *H. persicum* EO could be well-suited for industrial applications due to its widespread presence in Iran and Northern Europe, as well as its low price on the market (2–3 USD/kg).

### 2.3. Cytotoxicity Assay

Assessing the safety profile of insecticidal agents is crucial and often represents a limit for the registration and real-world application of novel insecticidal agents [50]. In this regard, the cytotoxicity of *H. persicum* EO and its main compounds was assessed on HEK293. As reported in Table 3, the concentration able to inhibit the cell growth by 50% (IC_50_) of the EO resulted in 100.2 ppm, while the main compounds showed a different cytotoxicity (IC_50_ value > 200 and 67.99 ppm for octyl acetate and hexyl butyrate, respectively).

Even if the EO was slightly less active than the pure compounds in the larvicidal assays, its lower cytotoxicity could favor its use. Although this is the first report on the cytotoxic activity on HEK cell lines, the EO has already been tested on diverse cancer cell lines such as human colon adenocarcinoma (LS180), human cervical adenocarcinoma (HeLa) and human B lymphoma (Raji) and was completely inactive at the concentrations tested [32]. Conversely, the EO was cytotoxic in the brine shrimp lethality test [51] with an LC_50_ of 0.0071 µL/mL. Regarding octyl acetate, this compound has been tested on different tumor cell lines, namely MDA-MB 231, T98G, A375, and HCT116, with IC_50_ values higher than 200 ppm [39]. The results herein reported could be the starting point to further investigate the safety of *H. persicum*-derived products for their exploitation in insecticide development.

## 3. Materials and Methods

### 3.1. Chemicals

The mixes of C_7_–C_40_ alkanes, hexyl butyrate, and octyl acetate were purchased from Merck (Milan, Italy). The *n*-hexane used for gas chromatography–mass spectrometry (GC-MS) analysis was acquired from Carlo Erba (Milan, Italy).

### 3.2. Plant Material

*H. persicum* schizocarps were collected from the Sahand mountains, Korde-deh, Maragheh, Iran (N 37°51′; E 46°43′, 2100–2200 m a.s.l), at full ripening stage (July 2023). The plant voucher specimen (codex no 4543) was deposited in the Herbarium of the Department of Horticultural Science, University of Maragheh, Iran.

### 3.3. Hydrodistillation

Hydrodistillation was performed on the dry plant material for 5 h employing a Clevenger-type apparatus. In detail, 1 kg of *H. persicum* schizocarps was placed into a 10 L Pirex distilling flask together with 7.3 L of distilled water and heated with a mantle system (Falc Instruments, Treviglio, Italy). The EO was collected by a Clevenger-type apparatus, separated from the aqueous phase and collected in a yield of 0.9% *w*/*w* on a dry weight basis. The EO was dried employing anhydrous sodium sulfate and kept in vials at 4 °C prior to analysis.

### 3.4. Chemical Characterization of the EO

The characterization of *H. persicum* EO was performed through an Agilent 8890 GC-MS. The detector was a single quadrupole, model 5977B, purchased from Agilent, Santa Clara, CA, USA. The instrument was equipped with an autosampler PAL RTC120 (CTC Analytics AG, Zwingen, Switzerland). The molecules, after separation in an HP-5MS capillary column (30 m, 250 µm i.d., 0.25 µm film thickness), were ionized by utilizing an electron ionization source (EI). The separation, identification and semi-quantification of the EO compounds were performed by using the same analytical conditions as those reported by Gugliuzzo et al. [52].

### 3.5. Mosquitoes

The laboratory-reared strains were as followed: *An. gambiae* G3 (MR4, MRA-112) was established in the insectary of the University of Camerino (Unicam) in 2022; the *Ae. albopictus* population was established in the Unicam insectary from field-collected mosquitoes from Petriolo (MC), Italy (43°13′15.75″ N; 13°27′56.73″ E), in 2018, while *Ae. aegypti* (New Orleans, LA, USA, 2011) was established in the Camerino insectary in 2020. The above-mentioned colonies were kept at 28 ± 2 °C and 80 ± 5% R.H., with a 12:12 h (L:D) photoperiod. Adults of *An. gambiae* were preserved with a 5% sucrose solution, while *Ae. albopictus* and *Ae. aegypti* with that at 10%. Mosquito larvae were grown in deionized water containing 0.5 g/L of artificial sea salt and nourished every day with a diet composed of a slurry of 2:2:1 bovine liver powder, tuna meal and Vanderzant vitamin mix [53].

### 3.6. Larvicidal Assays

Larvicidal assays were performed following the procedures of the World Health Organization (WHO) [54] applying some modifications [55]. Solutions of the EO and its two main constituents were prepared in dimethyl sulphoxide (DMSO) at a 1:10 ratio. The EO was tested at concentrations of 90, 100, 105, 110, and 115 ppm, while the pure compounds were tested at 70, 80, 90, 100, and 110 ppm. These concentrations were established after preliminary trials with different concentration ranges. For the assays, the initial solution was adequately diluted in 200 mL of distilled water in a 500 mL beaker. Afterwards, 25 third instar larvae were placed into each beaker and the trials were carried out in quadruplicate. Permethrin (Merck, Milan, Italy) and DMSO were the positive and negative controls, respectively. The LC_50_ values obtained for permethrin were consistent with those already reported in our previous work [56]. The assay was performed without giving food to the larvae and the mortality was monitored after 24 h. In detail, moribund and dead larvae were counted to calculate the mortality. Moribund larvae were those that did not respond to water movement and were unable to rise to the surface. Dead larvae were those that showed no movement when probed with a needle [54]. The assay was conducted at 28 ± 2 °C, 80 ± 5% R.H., with a 12:12 h (L:D) photoperiod.

### 3.7. MTT Cytotoxicity Assay

The HEK293 cell line was cultured in Eagle’s Minimum Essential Medium (MEM) with 2 mM L-glutamine, 100 IU/mL penicillin, 100 ppm streptomycin, and an addition of 10% heat-inactivated fetal bovine serum (HI-FBS). Cells were grown at 37 °C with 5% CO_2_ in humid atmosphere. The MTT [3-(4,5-dimethyl-2-thiazolyl)-2,5-diphenyl-2*H*-tetrazoliumbromide] assay was carried out to measure the relative cell viability [57]. The cells were seeded at a density of 2 × 10^4^ cells/mL. After 24 h, samples were treated with different concentrations of EO and pure compounds (0.78–400 ppm) solubilized in EtOH. Then, cells were left for 72 h in a humidified atmosphere with 5% CO_2_ at 37 °C. After this period, the MTT solution (5 mg/mL in phosphate-buffered saline, PBS) was added (10 µL) and the plates were left in incubation for 4 h at 37 °C. After the removal of the supernatant, DMSO was added to enable solubilization of the formazan crystals. The MTT reduction was assessed using a microplate spectrophotometer FLUOstar Omega working at 540 nm (BMG Labtech, Milan, Italy). Cisplatin (Merck, Milan, Italy) (0.01–50 ppm) was employed as a positive control. The experiments were performed three times. The cell survival curves were constructed after the comparison with the vehicle (EtOH). Cytotoxicity was expressed as IC_50_. The IC_50_ values were calculated using GraphPad Prism 5 program (GraphPad Software, San Diego, CA, USA).

### 3.8. Statistical Analysis

The quantification of the toxicity of the different products was calculated by probit analysis. The concentrations (ppm) of the tested products were log10 converted and the proportion of dead individuals after 24 h was used to calculate the LC_50_ and the LC_90_. For this purpose, the “ecotox” R package was used [58] to estimate the LC_50_ and LC_90_ with the corresponding 95% confidence interval and chi-squares. Then, a Generalized Linear Mixed Model was fit to test the efficacy of the products and different dosages, using the “glmmTMB” package [59]. As predictor variables, the percentage of mortality in terms of the number of dead individuals on total samples was used, leading to “1” when dead and “0” for alive. As such, a binomial distribution with replicate membership as a random factor was employed. Model fit was tested using the “DHARMa” package [60] and, next, the “car” package [61] to verify which factors of the model—i.e., species, products and concentrations—were significantly affecting the dependent variable. Afterwards, a post hoc analysis was performed using estimated marginal means with the Bonferroni correction, using the “emmeans” package [62], to eventually evaluate the statistical differences among different treatments and doses on the three mosquito species. The statistical analyses were performed in R 4.3.1 [63].

## 4. Conclusions

In recent years, the wide exploitation of synthetic insecticides has led to numerous environmental and health issues. In contrast, botanical insecticides offer a more sustainable alternative, potentially reducing the environmental impact of synthetic insecticides and limiting the spread of vectors of serious diseases. The results obtained in this study revealed that the EO obtained from *H. persicum* schizocarps and its two main constituents, octyl acetate and hexyl butyrate, exhibit significant larvicidal potential and warrant further investigation as mosquitocidal agents. Moreover, these three products showed minimal toxicity against the HEK293 cell line, suggesting their promise for practical applications. The broad diffusion of Golpar in Iran as well as in Northern Europe and its widespread use as a spice could facilitate its industrial application as an insecticidal product. Nevertheless, it is crucial to further investigate their specific mechanisms of action and assess their non-target toxicity to ensure safe and effective use.

## Figures and Tables

**Table 1:** Chemical composition of *Heracleum persicum* essential oil (EO).

No	Compound ^a^	LRI ^b^	RI Lit. ^c^	% ± SD ^d^	ID ^e^
1	*n*-hexanol	865	863	1.2 ± 0.0	Std
2	isopropyl-2-methyl butyrate	888	880	3.2 ± 0.0	RI, MS
3	isopropyl isovalerate	902	900	2.9 ± 0.0	RI, MS
4	isobutyl isobutyrate	912	908	0.2 ± 0.0	RI, MS
5	butyl isobutyrate	949	955	0.5 ± 0.0	Std
6	isobutyl butyrate	952	953	0.4 ± 0.0	RI, MS
7	isopropyl 3-methyl-2-butenoate	961	969	0.8 ± 0.0	RI, MS
8	butyl butanoate	994	993	1.6 ± 0.0	RI, MS
9	*n*-octanal	1001	998	1.3 ± 0.0	Std
10	hexyl acetate	1012	1007	1.1 ± 0.1	RI, MS
11	*ρ*-cymene	1021	1020	0.7 ± 0.0	Std
12	butyl 2-methyl butyrate	1040	1044	0.5 ± 0.0	RI, MS
13	butyl isovalerate	1045	1047	0.3 ± 0.0	RI, MS
14	*γ*-terpinene	1056	1054	0.2 ± 0.1	Std
15	2-methylbutyl butyrate	1057	1058	0.4 ± 0.0	RI, MS
16	*n*-octanol	1069	1063	3.4 ± 0.0	Std
17	linalool	1097	1095	1.2 ± 0.0	Std
18	hexyl isobutyrate	1148	1147	1.3 ± 0.0	RI, MS
19	hexyl butyrate	1191	1191	36.1 ± 0.2	Std
20	(*3Z*)-3-octenol acetate	1197	1190	3.9 ± 0.1	RI, MS
21	decyl aldehyde	1203	1204	0.6 ± 0.1	RI, MS
22	octyl acetate	1210	1214	23.7 ± 0.1	Std
23	hexyl 2-methyl butyrate	1236	1233	1.6 ± 0.0	RI, MS
24	hexyl isovalerate	1241	1241	0.2 ± 0.0	RI, MS
25	(*E*)-anethole	1282	1282	1.0 ± 0.0	Std
26	octyl isobutyrate	1344	1344	1.1 ± 0.0	RI, MS
27	hexyl hexanoate	1385	1382	1.2 ± 0.0	RI, MS
28	octyl butyrate	1388	1394	4.7 ± 0.1	RI, MS
29	octyl 2-methyl butyrate	1432	1434	1.9 ± 0.0	RI, MS
30	octyl hexanoate	1581	1575	0.4 ± 0.0	RI, MS
	Total identified (%)			97.4 ± 0.2	
	Grouped compounds (%)				
	Aliphatic compounds				
	Esters			87.9 ± 0.4	
	Aldehydes			0.6 ± 0.1	
	Alcohols			5.9 ± 0.0	
	Terpenes				
	Monoterpene hydrocarbons			0.9 ± 0.0	
	Oxygenated Monoterpenes			2.1 ± 0.1	

^a^ Compounds are reported according to the increasing order of their retention time by an HP-5MS capillary column. ^b^ LRI, linear retention index determined by injecting a homologous series of a mix of C_7_–C_40_ alkanes. ^c^ RI Lit., retention index reported from the literature. ^d^ Average of relative % area obtained from two independent analyses ± standard deviation (SD). ^e^ The methods employed to identify compounds were STD, achieved by comparing the mass spectrum with that of standard compounds; MS, obtained from comparison with WILEY275, ADAMS, FFSNC2, and NIST17 MS databases; and RI, by matching calculated LRI with those reported in ADAMS or NIST17 [28,29,30].

**Table 2:** Larvicidal activity of the *Heracleum persicum* essential oil (EO) and its main constituents against different mosquito species.

Product	Species ^a^	LC_50_ (95% CI ^c^) (ppm) ^b^	LC_90_ (95% CI ^c^) (ppm) ^b^	Intercept ± SE ^d^	Slope ± SE ^d^	χ^2^, *p*-Value
EO	*Ae. albopictus*	102.97 (101.26–104.62)	122.4 (118.66–128.02)	−34.37 ± 3.55	17.07 ± 1.76	11.410 *p* = 0.876
	*Ae. aegypti*	59.09 (55.92–62.27)	101.62 (93.43–113.42)	−9.64 ± 0.79	5.44 ± 0.44	6.639 *p* = 0.992
	*An. gambiae*	97.91 (95.88–99.60)	116.02 (113.14–120.17)	−34.62 ± 3.53	17.39 ± 1.75	8.640 *p* = 0.967
Hexyl butyrate	*Ae. albopictus*	85.40 (82.96–87.73)	113.65 (108.51–121.07)	−19.95 ± 1.84	10.33 ± 0.94	7.075 *p* = 0.989
	*Ae. aegypti*	53.59 (50.03–58.55)	99.49 (85.20–125.40)	−8.24 ± 0.83	4.76 ± 0.50	8.570 *p* = 0.968
	*An. gambiae*	70.97 (63.21–75.51)	116.48 (105.10–144.83)	−11.02 ± 2.22	5.95 ± 1.15	2.923 *p* = 0.999
Octyl acetate	*Ae. albopictus*	91.38 (88.92–94.00)	122.22 (115.83–131.75)	−19.89 ± 1.86	10.14 ± 0.95	10.610 *p* = 0.910
	*Ae. aegypti*	47.05 (44.44–50.26)	84.30 (74.56–100.35)	−8.46 ± 0.78	5.06 ± 0.48	10.759 *p* = 0.904
	*An. gambiae*	60.71 (34.71–71.89)	125.45 (111.10–182.90)	−7.25 ± 2.27	4.06 ± 1.15	7.782 *p* = 0.900

^a^ Species assayed in the study, *Aedes albopictus*, *Aedes aegypti* and *Anopheles gambiae*. ^b^ LC, lethal concentrations that kill 50% and 90% of exposed larvae, respectively. ^c^ 95% CI, lower and upper limits of the 95% confidence interval. ^d^ SE, standard error.

**Table 3:** Cytotoxic effect of *Heracleum persicum* essential oil (EO), octyl acetate, and hexyl butyrate on HEK293 cells.

	HEK293 ^a^ (IC_50_ ppm) ^b^
EO	100.2
95% CI ^c^	76.30–110.4
Octyl acetate	>200
95% CI	
Hexyl butyrate	67.99
95% CI	53.64–86.18
Positive control	
Cisplatin	3.92
95% CI	3.69 to 4.15

^a^ HEK293, human embryonic kidney cell line; ^b^ IC_50_, the concentration of compound that leads to a 50% reduction in cell growth (after 72 h of incubation); ^c^ CI, confidence interval.

## Data Availability

The datasets supporting the conclusions of this article are included within the article. The raw data supporting the conclusions of this article will be made available by the authors on request.
